# Are tri‐pronuclear embryos that show two normal‐sized pronuclei and additional smaller pronuclei useful for embryo transfer?

**DOI:** 10.1002/rmb2.12462

**Published:** 2022-05-23

**Authors:** Hiromi Takahashi, Rei Hirata, Junko Otsuki, Toshihiro Habara, Nobuyoshi Hayashi

**Affiliations:** ^1^ Okayama Couple’s Clinic Okayama Japan; ^2^ 12997 Assisted Reproductive Technology Center Okayama University Okayama Japan

**Keywords:** aneuploidy, embryo, ICSI, ploidy, tripronuclear zygote

## Abstract

**Purpose:**

This study aimed to analyze whether tripronuclear (3PN) zygotes, with two normal‐sized PNs and an additional smaller PN (2.1PN), can be used for embryo transfer.

**Method(s):**

A retrospective embryo cohort study was conducted on 695 patients who underwent intracytoplasmic sperm injection treatment. Blastocyst formation rates were compared between 2.1PN and 2PN zygotes and PGT‐A analysis was performed on 15 blastocysts derived from 2.1PN zygotes.

**Result(s):**

Blastocyst formation rates were comparable between 2.1PN (43.8%) and 2PN zygotes (54.8%; *p *= 0.212). The rates of blastocysts with good morphology derived from 2.1 PN and 2PN zygotes were 18.8% and 25.5%, respectively. No significant differences were detected (*p *= 0.383). All of the analyzed blastocysts were diploid; however, 13 of these were found to be aneuploid, with a further two being mosaic.

**Conclusion:**

Our results suggest that 2.1PN embryos can reach blastocyst stage. These blastocysts were diploid, however, predominantly aneuploid, and therefore could not be used for embryo transfer.

## Graphical abstract

Tri#x02010;pronuclear embryos that showing two normal‐sized pronuclei and additional smaller pronuclei are diploid but mostly aneuploidies and, therefore, may not be useful for embryo transfer.

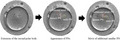

## 1 INTRODUCTION

In assisted reproductive technology (ART), fertilization is confirmed 16–18 h after insemination or intracytoplasmic sperm injection (ICSI). The formation of two pronuclei (PN) is considered normal fertilization. The two pronuclei of a normally fertilized zygote are generally equal in size and centrally located.^1^ If they are not of equal size, the resulting zygotes have little developmental potential^2^, ^3^ and a high degree of aneuploidy.^4^, ^5^, ^6^ Zygotes with one or more than three PNs are considered abnormally fertilized. In addition, zygotes that show three or more pronuclei (3PN and ≥4PN) are generally discarded regardless of their size.^7^ This is because embryos arising from zygotes with three PNs are considered to harbor a polyploid chromosomal constitution, and the transfer of these embryos is considered to result in a higher risk of miscarriage and/or molar pregnancy.^7^, ^8^, ^9^ It has been reported that blastocysts derived from 3PN zygotes, showing two normal‐sized PNs and additional smaller PN (2.1PN), are predominantly diploid and some of these have resulted in live births.^10^ According to their study, the 2.1PN‐derived blastocysts under study were also largely diploid (*n* = 12/14; 85.7%), while the remainder were triploid.

Various studies have shown that these tripronuclear human oocytes do not always develop into triploid embryos.^11^, ^12^ Although births derived from triploid zygotes have been reported, such infants generally do not live long after birth and only few have been reported as surviving for an unusual duration.^13^ Thus, it is important to analyze the chromosomal abnormalities of embryos to determine whether they are suitable for embryo transfer. Therefore, in this study, we aimed to investigate the developmental potential of 2.1PN embryos and analyze their ploidy and chromosomal abnormalities by performed preimplantation genetic testing for aneuploidy (PGT‐A).

## 2 MATERIALS AND METHODS

This retrospective study included 1001 cycles involving 695 patients who underwent ICSI treatment between November 2020 and July 2021. Thirty‐two 2.1PN zygotes were observed in 28 cycles from 26 patients. Written patient consent was obtained after providing a thorough explanation. This study was conducted with the approval of the ethics committee of Okayama Couple's Clinic (approval number 2020‐04).

### 2.1 Stimulation protocols

Ovarian stimulation, triggering final oocyte maturation, oocyte retrieval, fertilization, and embryo culture were conducted according to our standard protocols.^14^ The ovarian stimulation protocols were chosen depending on each patient's age and serum anti‐Müllerian hormone (AMH) level (Table 1). Patients in the long protocol group and the short protocol group were treated with a GnRH agonist (Buserelin acetate, Fuji Pharma). Treatment continued until the day on which 10 000 IU of human chorionic gonadotropins (hCG) (HCG, Fuji Pharma) was administered.

**TABLE 1:** Ovarian stimulation protocols

AMH level (ng/mL)/age (years)	<40	≧40
4 < AMH	PPOS, Antagonist	PPOS, Antagonist
1 < AMH ≦ 4	Antagonist, PPOS, Long	Antagonist, PPOS, Long, Short
0.5 < AMH ≦ 1	Antagonist, PPOS, Short, CC, AI	CC, AI, Short, Antagonist, PPOS
0.1 < AMH ≦ 0.5	Short, Antagonist, PPOS, CC, AI	CC, AI
AMH ≦ 0.1	CC, AI	CC, AI, Natural

For the long protocol, administration of the GnRH agonist commenced a week following their most recent ovulation. For the short protocol, administration of the GnRH agonist commenced on the second day of the menstrual cycle.

For the GnRH antagonist protocol, the GnRH antagonist (Cetrotide, Merck & Co) was administered when the leading follicle diameter reached 14–15 mm in diameter and was administered until the day of hCG administration. For all three long, short, and GnRH antagonist protocols, ovarian stimulation began on the third day of the cycle using hMG (Folyrmon‐P, Fuji Pharma; HMG TEIZO, ASKA Pharmaceutical; Ferring, Ferring Pharmaceuticals) or rFSH (Gonalef; Merck Serono), and continued until the day of hCG administration.

For the clomiphene citrate (CC) protocol, oral administration of 50 mg clomiphene citrate (Clomid; Fuji Pharma) was commenced at a rate of once a day on the third day of the menstrual cycle, and continued until the day of hCG administration. HMG was also administered until the day of hCG administration.

For the aromatase inhibitor (AI) protocol, oral administration of 5 mg AI (Letrozole “TEVA”; Pharmaceutical Industries Ltd) commenced at a rate of once a day on the third day of the menstrual cycle and was continued for 5 days.

Human Menopausal Gonadotrophin (HMG) was administered until the day of hCG administration. The starting dose for the progestin prime ovulation induction (PPOS) protocol was 2 mg/day of oral progestin (Lutoral, Fuji Pharma), administered orally once a day, on the third day of the menstrual cycle, and continuing until the day of hCG administration. HMG was administered until the day of hCG administration.

When at least two follicles reached a diameter of 18 mm or larger, 10 000 IU hCG was administered to induce ovulation.

### 2.2 Oocyte retrieval and denuding

Oocyte retrieval was performed 35.5 h after hCG administration, with transvaginal ultrasound‐guided aspiration. Oocytes were collected from the follicular fluid and washed in freshly equilibrated human tubal fluid medium with HEPES (Multipurpose Handling Medium; Irvine Scientific, CA, USA). They were then incubated in an insemination medium (NAKA medical inc, Tokyo, Japan) covered with mineral oil (Light Mineral Oil for Embryo Culture; Irvine, Cal., USA) for 2–4 h with 6.5% CO_2_/5% O_2_/88.5% N_2_ at 37°C in an incubator (MINC^™^, COOK MEDICAL INC, IN, USA).

The oocytes were denuded of the cumulus oophorus by a brief exposure to 40 IU/mL hyaluronidase (ICSI Cumulase; Cooper surgical, Trumbull, USA), and a pipette (STRIPPER; Cooper surgical, Trumbull, USA) was used to mechanically remove the oocytes from the surrounding cumulus cells.

Denuded oocytes were incubated in single‐step medium (ONE STEP Medium; NAKA medical inc, Tokyo, Japan) covered with mineral oil.

### 2.3 ICSI procedures and embryo culture

Intracytoplasmic sperm injection was performed on MII stage oocytes 39.5 h after hCG administration to induce ovulation.

Sperms were prepared using a density‐gradient centrifugation technique with Isolate (Irvine, Cal., USA) and the swim‐up method with insemination medium. The embryos were placed into the well‐of‐the‐well (WOW) culture system (LinKID^®^ micro25; DNP, Tokyo, Japan) using a single‐step medium and covered with mineral oil.

Fertilization was confirmed by the presence of two pronuclei 16–18 h after ICSI (Day 1). At the time of fertilization confirmation, the PNs diameter of 2PB3PN (a zygote with two polar bodies and three PNs) was measured, and zygotes that presented with two normal PNs and an additional smaller PN, not larger than one‐third the size of normal, were defined as 2.1PN.^10^

Cleavage stage embryos were evaluated based on Veeck's classification on the second day after oocyte retrieval.^15^ Blastocyst development was evaluated based on Gardner's classification on the fifth and sixth days after oocyte retrieval.^16^ Blastocyst formation with good morphology was defined as a grade of at least 3BB.

### 2.4 Blastocyst vitrification

The timing of cryopreservation depended entirely on blastocyst development. Blastocysts (grades 3–6) with good or fair ICM‐TE morphology (AA, AB, AC, BA, BB, BC, CA, and CB) were selectively cryopreserved on Day 5, except for those with poor ICM‐TE morphology (CC). Blastocysts not reaching this benchmark were given a further day in culture, after which they were cryopreserved with vitrification media (Cryotop; KITAZATO, Shizuoka, Japan) on Day 6.

### 2.5 Vitrified–thawed trophectoderm biopsies

All trophectoderm biopsies were processed after being vitrified–thawed for preimplantation genetic testing for aneuploidies (PGT‐A) via the PGTai^SM^ 2.0 technology platform (PGTai; Cooper Surgical, Trumbull, U.S.A.). Thawing procedures were performed with a thawing media (Cryotop; KITAZATO, Shizuoka, Japan). Laser‐assisted hatching was performed immediately after thawing. Blastocysts were immobilized with a holding pipet (COOK), and a hole of approximately 20 μm in the ZP was created with laser pulses (Saturn 5™ Active; Cooper surgical, Trumbull, USA). After opening the ZP, embryos were cultured in a time‐lapse incubator. When the blastocyst diameter reached approximately 160 µm, biopsies were performed as follows. TE cells were aspirated with a biopsy pipette, and after two to three laser pulses, the TE cells were removed by a quick flicking movement of the biopsy pipette against the holding pipette. A mean of five to eight TE cells were removed from blastocysts. TE cells were transferred to the PCR tubes following biopsy, after which the samples were sent to CooperGenomics.

### 2.6 Statistical analyses

We assessed continuous variables with a normal distribution and equal variances using Student's *t*‐tests. The difference in blastocyst formation rates was compared between 2.1PN and 2PN embryos with a chi‐squared test. Aneuploidy and ploidy analyses were evaluated by copy number variation (CNV) and single‐nucleotide polymorphisms (SNP).

## 3 RESULTS

Of the 7520 MII oocytes used for ICSI, 6070 (80.7%) were 2PN zygotes and 32 (0.4%) were 2.1PN zygotes (Table 2).

**TABLE 2:** Clinical outcomes of embryo development according to the fertilization classification

Fertilization classification	7520 MⅡ oocytes used for ICSI 379 degenerated oocytes					
	0PN	1PN	2PN	2.1PN	≥3PN^‡^	2cell
N. of embryos	583 (7.8%)	251 (3.3%)	6070 (80.7%)	32 (0.4%)	184^§^ (2.4%)	21 (0.3%)
N. of embryos which cultured until Day5^†^	107	218	6005	32	‐	21
N. of blastocyst formation on Day5	1 (0.9%)	14 (6.4%)	3288 (54.8%)	14 (43.8%)	‐	2 (9.5%)
N. of good morphology blastocyst formation on Day5	0	2 (0.9%)	1530 (25.5%)	6 (18.8%)		0
N. of blastocyst formation on Day6	0	15 (6.9%)	512 (8.5%)	2 (6.3%)	‐	0

^†^ Embryos transferred on Day3, embryos cryopreserved on Day3 and uncleaved embryos were excluded.

^‡^ ≥3PN other than 2.1PN.

^§^ ≥3PN embryos other than 2.1PN zygotes were not cultured after fertilization was confirmed.

Women in which 2.1PN zygotes occurred (*n* = 28 cycles) were on average 39.2 years of age, and those where 2.1PN zygotes did not occur (*n* = 973 cycle) were on average 37.3 years of age. There was a significant trend for the age of women to differ between the two groups (*p *= 0.067). The average age of men in which the occurrence of 2.1PN zygotes was observed was 41.8 years, which was significantly older than that of 38.7 years in which no occurrence of 2.1PN zygotes was observed (*p *= 0.012).

The average diameter and the area of male PNs before pronuclear membrane breakdown (PNMBD) in 2.1PN zygotes and 2PN zygotes were 27.8 µm versus 28.9 µm and 614.5 µm^2^ versus 661.1 µm^2^, respectively; no statistically significant differences were detected (*p *= 0.119, *p *= 0.150, respectively). However, the average diameter and the area of female PNs before PNMBD in 2.1PN zygotes (24.8 µm and 489.6 µm^2^, respectively) were significantly smaller than in 2PN zygotes (26.4 µm and 552.5 µm^2^, respectively; *p *= 0.014, *p *= 0.017, respectively). The average diameter and the area of the additional smaller PNs before PNMBD in 2.1PN zygotes were 11.9 µm and 117.2 µm^2^, respectively.

No statistically significant differences were found regarding Day 5 blastocyst formation rates between 2.1PN (43.8%) and 2PN zygotes (54.8%) (*p *= 0.212). In addition, Day 5 blastocyst formation rates with good morphology were 18.8% in 2.1PN zygotes and 25.5% in 2PN zygotes. No statistically significant difference was detected (*p *= 0.383) (Table 2). The same results were also found among patients with <40 years. No statistically significant differences were detected regarding Day 5 blastocyst formation rates between 2.1PN (60.0%) and 2PN zygotes (57.6%) (*p *= 0.877). Day 5 blastocyst formation rates with good morphology were 20.0% and 28.0%, respectively, and no statistically significant difference was detected (*p *= 0.573) (Table 3).

**TABLE 3:** Clinical outcomes of embryo development according to the fertilization classification in women younger than age 40 years

Fertilization classification	5806 MⅡ oocytes used for ICSI 274 degenerated oocytes					
	0PN	1PN	2PN	2.1PN	≥3PN^‡^	2cell
N. of embryos	461 (7.9%)	188 (3.2%)	4733 (81.5%)	10 (0.2%)	126^§^ (2.2%)	14 (0.2%)
N. of embryos which cultured until Day5^†^	87	161	4700	10	‐	21
N. of blastocyst formation on Day5	1 (1.1%)	12 (7.5%)	2706 (57.6%)	6 (60.0%)	‐	2 (9.5%)
N. of good morphology blastocyst formation on Day5	0	2 (1.2%)	1317 (28.0%)	2 (20.0%)	‐	0
N. of blastocyst formation on Day6	0	11 (6.8%)	386 (8.2%)	0	‐	0

^†^ Embryos transferred on Day3, embryos cryopreserved on Day3 and uncleaved embryos were excluded.

^‡^ ≥3PN other than 2.1PN.

^§^ ≥3PN embryos other than 2.1PN zygotes were not cultured after fertilization was confirmed.

Preimplantation genetic testing for aneuploidy analysis was performed on 15 blastocysts derived from 2.1PN zygotes. All of the analyzed blastocysts were diploid; however, 13 of these were found to be aneuploid, with a further two being mosaic (Table 4).

**TABLE 4:** Clinical outcomes of PGT‐A

Sample ID	Ovarian stimulation	Case of infertility	Female age (y)	Male age (y)	Sample Stage (Day)	Blastocyst grade	Result	Chromosomes impacted	Interpretation	Ploidy analysis (SNP)
10	Antagonist	Male factor	29	30	5	CB	Aneuploid	−3,del(5)(q35.2‐qter) [mos]	Abnormal	Diploid
1	Long	Male factor	30	26	5	AA	Mosaic	del(7)(pter‐p11.2) [mos]	Low Level Mosaic	Diploid
2	PPOS	Male factor, Endometriosis	31	31	5	BB	Aneuploid	+5,+15	Abnormal	Diploid
12	PPOS	Male factor	31	32	5	BB	Aneuploid	+4,−6 [mos],−8 [mos]	Complex Abnormal	Diploid
14	PPOS	Male factor	31	32	5	BC	Aneuploid	−1 [mos],−6,+18	Complex Abnormal	Diploid
8	Long	Male factor	32	38	6	BA	Mosaic	dup(1)(q31.3‐qter) [mos]	High Level Mosaic	Diploid
3	Antagonist	Cause unknown	41	41	5	BA	Aneuploid	+3	Abnormal	Diploid
4	Short	Male factor	41	42	5	BB	Aneuploid	dup(1)(q12‐qter) [mos],+17,+22	Complex Abnormal	Diploid
5	Short	Cause unknown	41	34	6	BB	Aneuploid	+10	Abnormal	Diploid
13	CC	Fallopian tube factor	42	42	5	BC	Aneuploid	−7,+14,+16 [mos],−21,+X	Complex Abnormal	Diploid
9	CC	Male factor	43	42	6	BA	Aneuploid	−22	Abnormal	Diploid
11	CC	Male factor	43	53	6	BC	Aneuploid	−11,+21	Abnormal	Diploid
6	AI	Cause unknown	45	43	5	AB	Aneuploid	−13,−16	Abnormal	Diploid
7	AI	Cause unknown	45	43	5	BC	Aneuploid	+10 [mos],+16	Abnormal	Diploid
15	Antagonist	Immune factor	47	50	7	BC	Aneuploid	−2,del(3)(pter‐p22.3),+7 [mos],−8,dup(11)(pter‐p15.1) [mos],+12 [mos],+15,−21,+22 [mos]	Complex Abnormal	Diploid

The comparison of PGT‐A results between 2.1PN‐derived and 2PN‐derived embryos before and after propensity score matching are shown in Table S1. Note that the sample sizes for the comparison are smaller than the standard sizes.

## 4 DISCUSSION

In this study, we investigated whether 2.1PN embryos are useful for embryo transfer by analyzing ploidies and aneuploidies of 2.1PN‐derived blastocysts. We found that 2.1PN‐derived blastocysts were diploids; however, they were predominantly aneuploid.

According to Rosenbusch et al., there are three possible causes for the appearance of three or more PNs in ICSI. Firstly, complete nonextrusion of the second polar body causes the formation of an additional haploid female PN. In which case, the one‐cell zygote would be triploid. Secondly, the second PB is extruded but does not include all 23 chromatids (incomplete maternal chromatid segregation). This would lead to an additional hypohaploid female PN and a hypotriploid zygote. Finally, the second PB is extruded, but the remaining 23 oocyte chromatids are dispersed, giving rise to two hypohaploid maternal PNs.^8^ This final case is considered to be a diploid 3PN zygote.

**FIGURE 1:**
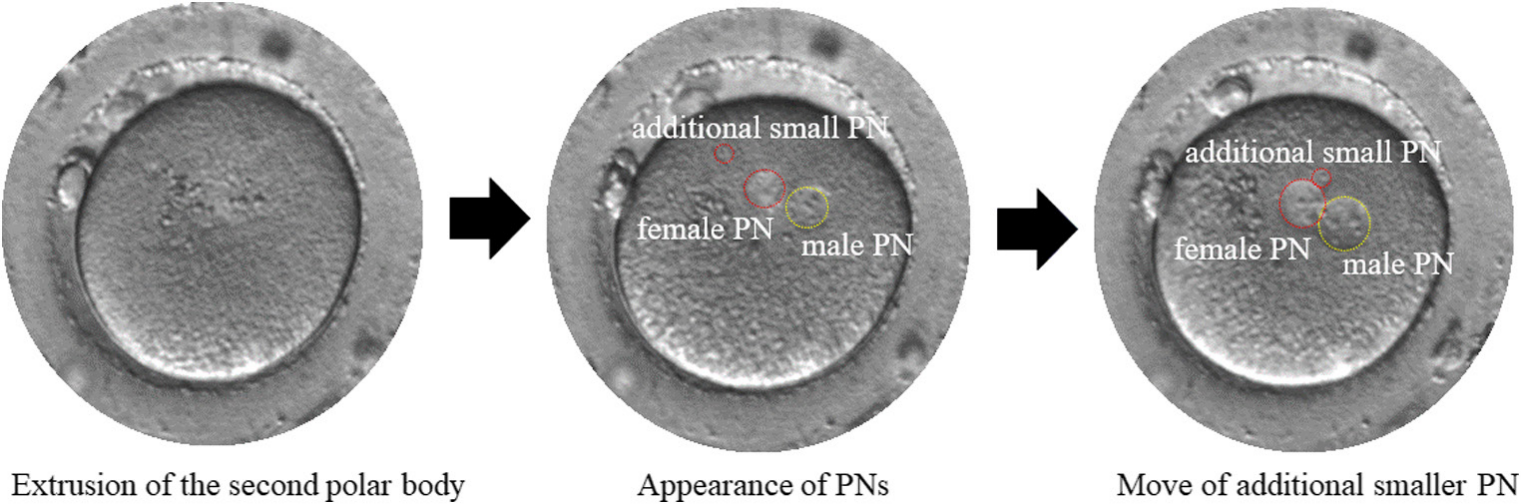
The movement of additional smaller PN. The additional smaller PN was formed later than the female PN and always developed right below the second polar body, prior to moving toward the male PN

In this study, by confirming extrusion of the second polar body in the ICSI cycle, the possibility of triploids due to complete nonextrusion of the second polar body (case one above) was excluded, and 2.1PN zygotes were analyzed. Although the mechanisms of 2.1PN formation are not yet fully understood, this study confirmed that the additional smaller PN was formed later than the female PN and always developed right below the second polar body, prior to moving toward the male PN (Figure 1, Movie S1). Furthermore, there was no difference in the size of male PNs between 2PN and 2.1PN. However, the female PNs of 2.1PNs were smaller than those of 2PNs. This suggests that the additional smaller PNs may be derived from the lagging chromosomes, which appear during chromosome segregation at the time of the second polar body extrusion. Therefore, the development of 2.1PN embryos is explained by case two (described above), and its polyploidy is considered diploid.

In fact, 2.1PN and 2PN zygotes developed to the blastocyst stage with a comparable probability in this study and all blastocysts from 2.1PN‐derived embryos upon which PGT‐A analysis was performed were diploid. However, most of these were aneuploid and unsuitable for embryo transfer. Two of the blastocysts from 2.1PN‐derived embryos upon which PGT‐A analysis was performed were mosaics. One was a low‐level mosaic (20%–40% mosaic) and the other was a high‐level mosaic (40%–80% mosaic).

The reproductive potential of mosaic embryos remains controversial.^17^, ^18^, ^19^ Our study suggests that 2.1PN‐derived embryos may not be suitable for transfer. However, in situations when no other embryos are available for transfer, patients should be adequately informed regarding the low normality rate of 2.1 PN zygotes prior to giving their consent, and transferring embryos derived from 2.1PN zygotes. Such transfers should be performed with caution. PGT‐A is an option for confirming the normality of 2.1 PN zygotes.

As the occurrence of 2.1PN is rare, only a limited number of 2.1PN are available for analysis. Thus, multicenter analysis will be required to investigate confounding factors such as patients’ age. Nevertheless, in this study, patients’ ages in the cycles with 2.1PN zygotes were higher than those without 2.1PN zygotes, and the two patients with mosaic embryos were relatively young (30 and 32 years). Although it has been reported that chromosomal aneuploidy is associated with age,^20^, ^21^, ^22^, ^23^ further research is required to gain a more complete understanding of whether the aneuploidy of 2.1PN embryos is associated with patient age.

## CONFLICT OF INTEREST

Hiromi Takahashi, Rei Hirata, Junko Otsuki, Toshihiro Habara and Nobuyoshi Hayashi declare that they have no conflicts of interest.

## ETHICAL APPROVAL

Approval was obtained from the ethics committee of Okayama Couple's Clinic (approval number: 2020–04). The procedures used in this study adhere to the tenets of the declaration of Helsinki.

## HUMAN RIGHTS STATEMENTS AND INFORMED CONSENT

All patients were well informed and written informed consent was obtained prior to the treatment period.

## ANIMAL STUDIES

This article does not contain any experimental studies with animal subjects on the part of any of the authors.

## Supporting information

Table S1

Video S1
