# Supplementary material for: Are tri‐pronuclear embryos that show two normal‐sized pronuclei and additional smaller pronuclei useful for embryo transfer?
Source: Reprod Med Biol. 2022 May 23;21(1):e12462. doi: 10.1002/rmb2.12462 (PMC9126567; doi:10.1002/rmb2.12462)
Supplement: Supplementary file 1 — Table S1 [file RMB2-21-e12462-s002.doc]

|  | Before matching | | | | After matching | | | |
| --- | --- | --- | --- | --- | --- | --- | --- | --- |
|  | 2.1PN-derived embryos | 2PN-derived embryos^†^ | p-value | SMD | 2.1PN-derived embryos | 2PN-derived embryos | p-value | SMD |
| # of embryos | 15 | 79 |  |  | 13 | 13 |  |  |
| Female age | 38.1 | 38.0 | 0.882 | 0.035 | 36.9 | 37.1 | 0.949 | 0.026 |
| Euploid embryos (%) | 0 (0/15) | 29.1 (23/79) | 0.016 |  | 0 (0/13) | 30.8 (4/13) | 0.030 |  |
| Mosaic embryos (%) | 13.3 (2/15) | 16.5 (13/79) | 0.762 |  | 15.4 (2/13) | 7.7 (1/13) | 0.539 |  |
| Aneuploid embryos (%) | 86.7 (13/15) | 54.4 (43/79) | 0.020 |  | 84.6 (11/13) | 61.5 (8/13) | 0.185 |  |

Supplementary Table 1. Comparison of PGT-A results between 2.1PN-derived and 2PN-derived embryos before and after propensity score matching

^†^During the study period, 79 blastocysts derived from 2PN were subjected to PGT-A.

SMD: Standardized Mean Difference. The SMD is considered to be balanced when it is less than 0.1.

A chi-squared test was used to compare 2.1PN-derived and 2PN-derived embryos with respect to PGT-A results. Propensity score matching was conducted to adjust female age as a possible confounder.
